# Supplementary material for: Regulation of TRI5 expression and deoxynivalenol biosynthesis by a long non-coding RNA in Fusarium graminearum
Source: Nat Commun. 2024 Feb 9;15:1216. doi: 10.1038/s41467-024-45502-w (PMC10853542; doi:10.1038/s41467-024-45502-w)
Supplement: Supplementary file 1 — Supplementary Information [file 41467_2024_45502_MOESM1_ESM.pdf]

Supplementary Information

Regulation of TRI5 expression and deoxynivalenol biosynthesis by a long non-coding RNA in *Fusarium graminearum*

Panpan Huang[1,2], Xiao Yu[1], Huiquan Liu[1], Mingyu Ding[1], Zeyi Wang[2], Jin-Rong Xu[2]*, and Cong Jiang[1]*

[1] State Key Laboratory for Crop Stress Resistance and High-Efficiency Production, College of Plant Protection, Northwest A&F University, Yangling, Shaanxi 712100, China.

[2] Dept. of Botany and Plant Pathology, Purdue University, West Lafayette, IN 47907. USA.

Contents:

I. Supplementary Figure 1-7

II. Supplementary Table 1-3

I. Supplementary Figures

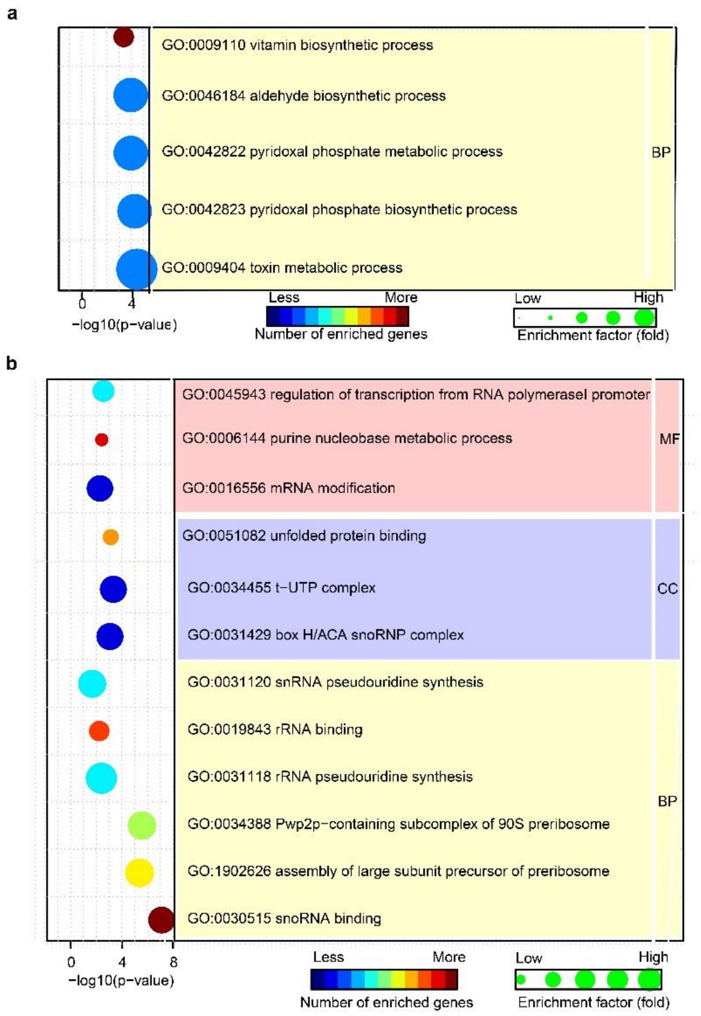

Supplementary Fig. 1 GO enrichment analysis with DEGs in the *tri6* and *tri10* mutants.

a Enriched gene ontology (GO) terms in genes with down-regulated transcripts in the *tri6* mutant. **b** Enriched gene ontology (GO) terms in genes with down-regulated transcripts in the *tri10* mutant. MF, molecular function; CC, cell component; BP, biological process.

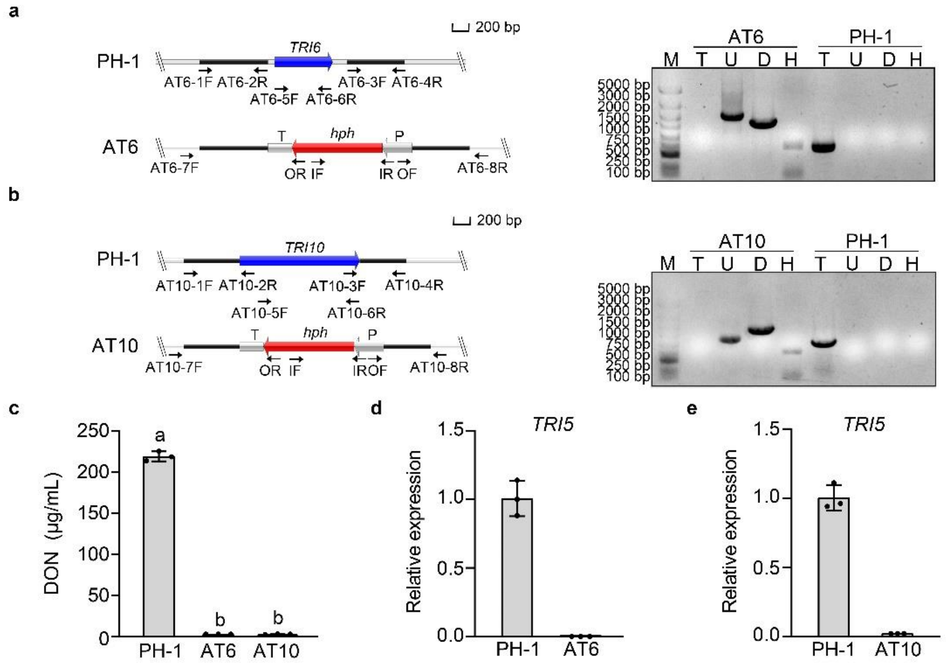

Supplementary Fig. 2 Generation of the *tri6* and *tri10* gene replacement mutants by an inverted *hph* cassette.

a Schematic drawing of the primers used to generate gene replacement transformants AT6 and screen for knockout mutants (left panel). The upstream and downstream flanking sequences of the *TRI6* gene were amplified with primer pairs AT6-1F/AT6-2R and AT6-3F/AT6-4R and connected to the inverted *hph* cassette to generate gene replacement constructs. P, TrpC promoter; T, CaMV ployA signal terminator. DNA isolated from the wild-type strain PH-1 and *tri6* mutant (AT6) for the PCR verification of *TRI6* deletion (right panel). PCR products amplified by primer pairs AT6-7F/OR (lane U) and OF/AT6-8R (lane D) showed the occurrence of homologous recombination at the upstream and downstream flanking sequences of the *TRI6* gene, respectively. Lane T was amplified with primer pairs AT6-5F and AT6-6R to show the deletion of *TRI6* genes. Lane H showed the amplification of the inverted *hph* gene with primers IF and IR. M, molecular marker. **b** Schematic drawing of the primers used to generate gene replacement transformants AT10 and screen for knockout mutants (left panel). The upstream and downstream flanking sequences of the *TRI10* gene were amplified with primer pairs AT10-1F/AT10-2R and AT10-3F/AT10-4R and connected to the inverted *hph* cassette to generate gene replacement constructs. DNA isolated from the wild-type strain PH-1 and *tri10* mutant (AT10) for the PCR verification of *TRI10* deletion (right panel). PCR products amplified by primer pairs AT10-7F/OR (lane U) and OF/AT10-8R (lane D) showed the occurrence of homologous recombination at the upstream and downstream flanking sequences of the *TRI10* gene, respectively. Lane T was amplified with primer pairs AT10-5F and AT10-6R to show the deletion of *TRI10* genes. Lane H showed the amplification of the inverted *hph* gene with primers IF and IR. M, molecular marker. **c** Deoxynivalenol (DON) levels in 7-day-old LTB cultures of wild-type

PH-1, AT6, and AT10 transformants. **d** Assayed for the expression of *TRI5* by qRT-PCR with RNA isolated from 3-day-old LTB cultures of the wild-type PH-1 and AT6. **e** Assayed for the expression of *TRI5* by qRT-PCR with RNA isolated from 3-day-old LTB cultures of the wild-type PH-1 and AT10. For each gene, its expression level in PH-1 was arbitrarily set to 1. For **c**, **d**, and **e**, mean and standard deviation were estimated with data from three ($n = 3$) independent replicates (marked with black dots on the bars). For DON production, different letters indicate significant differences based on the one-way ANOVA followed by Turkey's multiple range test. Differences were considered statistically significant when p-value is < 0.05. The exact p-values are shown in the Source Data file.

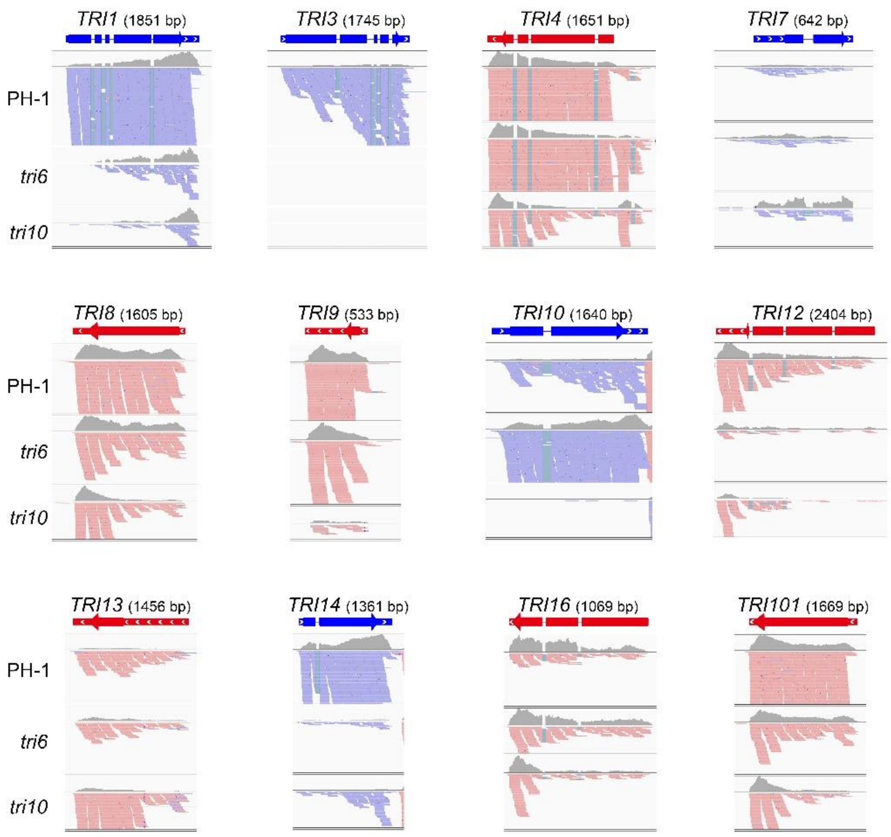

Supplementary Fig. 3 Expression profiles of the *TRI* genes without antisense transcripts. Predicted ORFs of marked *TRI* genes and read counts of their transcripts in RNA-seq data of PH-1 and the *tri6* and *tri10* deletion mutants. No antisense transcripts of these *TRI* genes were observed. The length of transcripts was indicated in the figure.

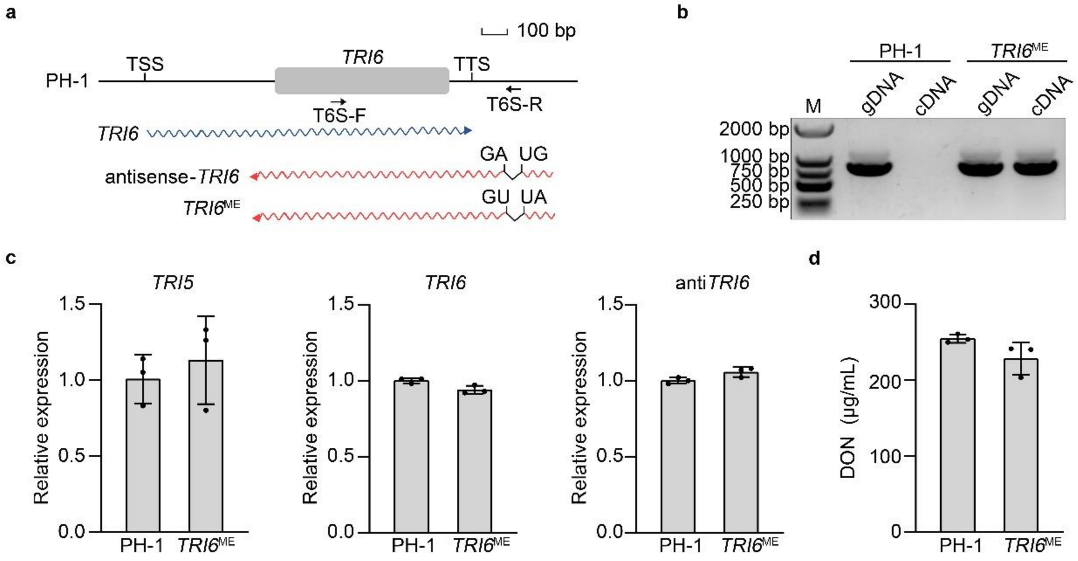

Supplementary Fig. 4 The antisense transcripts of *TRI6*.

a Schematic drawing of the *TRI6* and *TRI6*^{ME} alleles and its sense and antisense transcripts. Specific primers for PCR verification were indicated. **b** Verification by PCR through specific primer T6S-F/T6S-R. RNA isolated from 3-day-old LTB cultures of PH-1 was used for cDNA synthesis. Mutations on the splicing site disrupted the intron splicing in the *TRI6*^{ME} strain. M, molecular marker. **c** Relative expression levels of *TRI5*, *TRI6*, and antisense-*TRI6* were assayed by qRT-PCR with RNA isolated from 3-day-old LTB cultures of PH-1 and *TRI6*^{ME} transformant. For each gene, its expression level in PH-1 was arbitrarily set to 1. **d** DON production in 7-day-old LTB cultures of PH-1 and *TRI6*^{ME} transformant. For **c** and **d**, mean and standard deviation were estimated with data from three (n = 3) independent replicates (marked with black dots on the bars). For DON production, no significant statistical differences were observed based on the two-tailed unpaired t test. The exact p-values are shown in the Source Data file.

UAGAUCGUACAGCUAAAUUCAGAGCCUCCUGCUAAACCUAAAAGGGCUAAGUUGCCCAAACCUUAUUCAA
CGCAGUUGCAGUGCAUUCGGGAGCCAGCUCCGCGGGAUAUAUGUGAUGGCCGGCAGUUUCGUAGUGCU
GAUCAUAAAAGUGGUCAUAUUUAAGGCCUGUCGCCCAGGUAGACUUUUGCCAGGGCACAAUAUAACACCG
CGGCUACCUAGGUAAGUGAGGCUUUCUUCUGUGUUGAUAGAUGGUCGUUUCACUAGUCCAAACACAGAC
CACGGACAACGACCGAACUCAAUAUCCCGAUCCAAGGAUUGAUCCCUAGAUUUAGGCCUACUCACGGCCC
UUUGAUACUAGCAUCUAGCACCAAUCGCUUGUGUAAGUCUACCAAGCGUGUCGAGCUAAAGACAAAAUGA
ACCAAGAGUUUGUUCCAAGAGCCGGAUGUUUUCUGAUAUCUGU AGCCUUGCAGGGAAUGAGAGAGCACG
UCCAUACGUCAAGGUCUCUCUUCACGACUGUCUGGUUGGGGACGCUAUUCGCAUUGACUUUGGAUCAGU
CUUUAGGCCUAACAAUACAAUCUUGACUAACAACUGCAUACGGGUCAAGAUAUUUGCCAUCUAAUGAUAU
AUAGUUAAUAGCAACAGAACUUUGUAAUUAGAGAAUGACAACGACUUGAC

Supplementary Fig. 5 Full-length transcript of RNA5P.

Two isoforms of RNA5P are formed due to alternative splicing of the intron and are predicted to encode two small proteins that share the same stop codon. Two isoforms of RNA5P were underlined in red and blue, respectively. The GU-AG splicing sites were indicated with red font. The red arrow marks 3×FLAG.

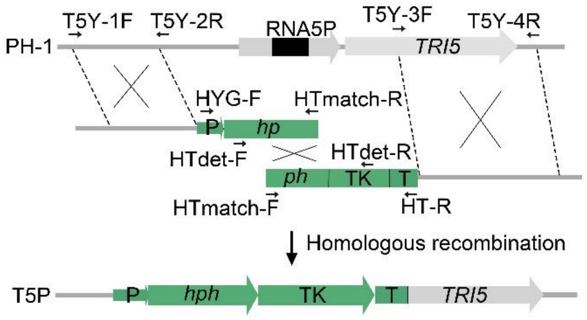

Supplementary Fig. 6 Schematic drawing of the primers used to generate gene replacement transformants T5P (the *TRI5*$^{\Delta\text{promoter}}$ mutant).

The upstream and downstream flanking sequences of the *TRI5* gene were amplified with labelled primer pairs and connected to the hygromycin phosphotransferase (*hph*)-thymidine kinase (TK) fusion cassette to generate the *TRI5*$^{\Delta\text{promoter}}$ mutant (T5P). P, TrpC promoter; T, CaMV ployA signal terminator.

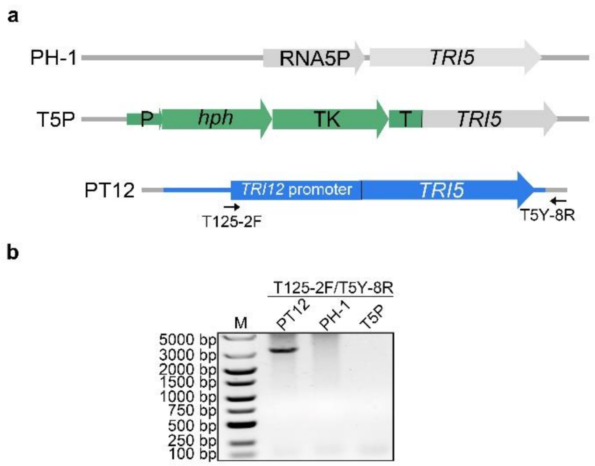

Supplementary Fig. 7 PCR verification of the P_{TRI12}-*TRI5* transformants.

a Schematic drawing of RNA5P in the upstream region of *TRI5* and the generation of the *TRI5*$^{\Delta promoter}$ (T5P) and P_{TRI12}-*TRI5* (PT12) transformants. **b** PCR products were amplified with primer pairs T125-2F and T5Y-8R from genomic DNA isolated from the wild-type strain PH-1, T5P (*TRI5*$^{\Delta promoter}$ transformant), and PT12 (P_{TRI12}-*TRI5* transformant). PCR products amplified from the DNA templates of PT12 transformant showed the replacement of the *TRI5* promoter with that of *TRI12*. DNA template isolated from the wild-type PH-1 (WT) and T5P (*TRI5*$^{\Delta promoter}$ transformant) was used as a control. M, molecular marker.

II. Supplementary Tables

Supplementary Table 1. Strains of *Fusarium graminearum* used or generated in this study.

Strains	Brief description	References
PH-1	Wild-type strain of *F. graminearum*	(Cuomo et al., 2007)
tri6	The *tri6* deletion mutant of PH-1	(Seong et al., 2009)
tri10	The *tri10* deletion mutant of PH-1	(Seong et al., 2009)
TRI6^{AD}	Deletion of the promoter of anti*TRI6* in PH-1	This study
tri5	The *tri5* deletion mutant of PH-1	This study
RAT1	P_{RP27}-anti*TRI5* transformant of PH-1	This study
RAT2	P_{RP27}-anti*TRI5* transformant of PH-1	This study
L3F	RNA5P-3×FLAG-T_{CaWV} transformant of PH-1	This study
EF1	*ESA1*-3×FLAG transformant of PH-1	(Jiang et al., 2020)
T5P	Deletion of the promoter of *TRI5* in PH-1	This study
RNA5P^{M}	Transformant with a nucleotide inserted in the RNA5P	This study
PT12	P_{TRI12}-*TRI5* transformant of T5P	This study
LT7	P_{RP27}-RNA5P-T_{CaWV} transformant of PH-1	This study
LT10	P_{RP27}-RNA5P-T_{CaWV} transformant of PH-1	This study
TR1	P_{TrpC}-RNA5P transformant of T5P	This study
TR2	P_{TrpC}-RNA5P transformant of T5P	This study
ATR1	$P_{TrpC\text{-}inverted}$-RNA5P transformant of T5P	This study
ATR3	$P_{TrpC\text{-}inverted}$-RNA5P transformant of T5P	This study
TM6	Transformant with GTGA to TGAG mutation in the promoter of RNA5P	This study
TM66	Transformant with GTGA to TGAG mutation in the promoter of RNA5P	This study
AT6	The *tri6* deletion mutant of PH-1 with the replacement by reverse-transcribed *hph* cassette	This study
AT10	The *tri10* deletion mutant of PH-1 with the replacement by reverse-transcribed *hph* cassette	This study
TRI6^{ME}	Transformant of *TRI6*^{AD} with the mutation on the splicing sites of *antiTRI6*	This study

Supplementary Table 2. DEGs up- or down-regulated in both the *tri6* and *tri10* mutants.

Up-regulated DEGs	
**Gene ID**	**Annotation**
FG1G13350	Hypothetical protein
FG1G17480	Hypothetical protein
FG1G36450	Nik-1 protein
FG1G36460	Isoprenylcysteine carboxyl methyltransferase
FG1G50780	Hypothetical protein
FG2G10860	Hypothetical protein
FG2G12010	Glucose-repressible protein Grg1
FG2G12090	MFS transporter
FG2G13970	Cryptochrome DASH
FG3G19710	Extracellular serine-rich protein
FG3G21560	Heam oxygenase-like protein
FG3G30990	Kes1 protein
FG4G20830	Allergen Fus c 3 protein
FG4G23950	Hypothetical protein

Down-regulated DEGs	
**Gene ID**	**Annotation**
FG1G00280	O-methylsterigmatocystin oxidoreductase
FG1G01160	*TRI1*
FG1G02270	Hypothetical protein
FG2G09410	Phosphoenolpyruvate carboxykinase
FG2G20970	Aminoglycoside phosphotransferase protein
FG2G26160	*TRI3*
FG2G26190	*TRI5*
FG2G26220	*TRI11*
FG2G26240	*TRI12*
FG2G26260	*TRI14*
FG3G00040	Integral membrane protein PTH11
FG3G04840	Pyridoxine biosynthesis protein PDX1
FG3G04850	Glutamine amidotransferase
FG4G08380	Aurora protein kinase
FG4G21810	Hypothetical protein
FG4G22230	Omega-6 fatty acid desaturase
FG4G22330	*TRI101*

Supplementary Table 3. The TPM (Transcripts Per Million) values of RNA5P, *TRI5*, anti*TRI5*, *TRI6*, anti*TRI6*, and a housekeeping gene *ACTIN* in PH-1.

RNA5P	*TRI5*	anti*TRI5*	*TRI6*	anti*TRI6*	*ACTIN*
1.68	175.55	3.81	27.92	8.66	203.98
