# Supplementary material for: Regulation of TRI5 expression and deoxynivalenol biosynthesis by a long non-coding RNA in Fusarium graminearum
Source: Nat Commun. 2024 Feb 9;15:1216. doi: 10.1038/s41467-024-45502-w (PMC10853542; doi:10.1038/s41467-024-45502-w)
Supplement: Supplementary file 3 — Description of Additional Supplementary Files [file 41467_2024_45502_MOESM3_ESM.pdf]

Description of Additional Supplementary Files:

Supplementary Data 1: Differentially Expressed Genes (DEGs) up- and downregulated in the tri6 mutant.

Supplementary Data 2: Differentially Expressed Genes (DEGs) up- and down-regulated in the tri10 mutant.

Supplementary Data 3: PCR primers used in this study.
