# Supplementary material for: Regulation of TRI5 expression and deoxynivalenol biosynthesis by a long non-coding RNA in Fusarium graminearum
Source: Nat Commun. 2024 Feb 9;15:1216. doi: 10.1038/s41467-024-45502-w (PMC10853542; doi:10.1038/s41467-024-45502-w)
Supplement: Supplementary file 7 — Reporting Summary [file 41467_2024_45502_MOESM7_ESM.pdf]

nature portfolio

Corresponding author(s): Jin-Rong Xu

Last updated by author(s): Jan 16, 2024

Reporting Summary

Nature Portfolio wishes to improve the reproducibility of the work that we publish. This form provides structure for consistency and transparency in reporting. For further information on Nature Portfolio policies, see our Editorial Policies and the Editorial Policy Checklist.

Statistics

For all statistical analyses, confirm that the following items are present in the figure legend, table legend, main text, or Methods section.

n/a	Confirmed	
☐	☒	The exact sample size (n) for each experimental group/condition, given as a discrete number and unit of measurement
☐	☒	A statement on whether measurements were taken from distinct samples or whether the same sample was measured repeatedly
☐	☒	The statistical test(s) used AND whether they are one- or two-sided<br>*Only common tests should be described solely by name; describe more complex techniques in the Methods section.*
☒	☐	A description of all covariates tested
☒	☐	A description of any assumptions or corrections, such as tests of normality and adjustment for multiple comparisons
☐	☒	A full description of the statistical parameters including central tendency (e.g. means) or other basic estimates (e.g. regression coefficient) AND variation (e.g. standard deviation) or associated estimates of uncertainty (e.g. confidence intervals)
☐	☒	For null hypothesis testing, the test statistic (e.g. F, t, r) with confidence intervals, effect sizes, degrees of freedom and P value noted<br>*Give P values as exact values whenever suitable.*
☒	☐	For Bayesian analysis, information on the choice of priors and Markov chain Monte Carlo settings
☒	☐	For hierarchical and complex designs, identification of the appropriate level for tests and full reporting of outcomes
☒	☐	Estimates of effect sizes (e.g. Cohen's d, Pearson's r), indicating how they were calculated

Our web collection on statistics for biologists contains articles on many of the points above.

Software and code

Policy information about availability of computer code

Data collection	Strand-specific RNA-seq libraries were sequenced with Illumina HiSeq 2500 at Novogene Bioinformatics Technology (China). Quantitative reverse transcription polymerase chain reaction (qRT-PCR) assays were performed using the CFX96 Real-Time System (Bio-RAD, USA).
Data analysis	The resulting RNA-seq reads were mapped onto the updated version (Named YL1) of reference genome of F. graminearum strain PH-1 by HISAT2 and visualized with the Integrative Genomics Viewer (IGV) tool (software.broadinstitute.org/software/igv/). The number of reads (count) mapped to each gene were calculated by feature Counts. Genes with log2FC greater than 1 and FDR less than 0.05 were identified as Differentially Expressed Genes (DEGs) using the edge Run package with the exact Test function. GO enrichment analysis was analyzed with Blast2GO. Graph pad Prism 8.0 for statistical analysis and data representation.

For manuscripts utilizing custom algorithms or software that are central to the research but not yet described in published literature, software must be made available to editors and reviewers. We strongly encourage code deposition in a community repository (e.g. GitHub). See the Nature Portfolio guidelines for submitting code & software for further information.

Data

Policy information about availability of data

All manuscripts must include a data availability statement. This statement should provide the following information, where applicable:

- Accession codes, unique identifiers, or web links for publicly available datasets
- A description of any restrictions on data availability
- For clinical datasets or third party data, please ensure that the statement adheres to our policy

Data supporting the major findings of this work are available within the paper and its Supplementary Information files. The RNA-seq data generated in this study were have been deposited in the NCBI Sequence Read Archive database under the accession code of PRJNA1044545 (https://www.ncbi.nlm.nih.gov/bioproject/PRJNA1044545). The reference genome of F. graminearum strain PH-1 used in this study are available in the NCBI GenBank database under accession code PRJNA782099 (https://www.ncbi.nlm.nih.gov/bioproject/?term=PRJNA782099). Source data are provided with this paper.

Research involving human participants, their data, or biological material

Policy information about studies with human participants or human data. See also policy information about sex, gender (identity/presentation), and sexual orientation and race, ethnicity and racism.

Reporting on sex and gender	N/A
Reporting on race, ethnicity, or other socially relevant groupings	N/A
Population characteristics	N/A
Recruitment	N/A
Ethics oversight	N/A

Note that full information on the approval of the study protocol must also be provided in the manuscript.

Field-specific reporting

Please select the one below that is the best fit for your research. If you are not sure, read the appropriate sections before making your selection.

☒ Life sciences ☐ Behavioural & social sciences ☐ Ecological, evolutionary & environmental sciences

For a reference copy of the document with all sections, see nature.com/documents/nr-reporting-summary-flat.pdf

Life sciences study design

All studies must disclose on these points even when the disclosure is negative.

Sample size	No statistical methods were used to determine sample size. The specific sample size for each experiment is delineated in the methods section and Figure legends.
Data exclusions	No data were excluded from the analyses.
Replication	All attempts at replication were successful. The number of replicates is indicated in the corresponding figure legend and/or in the corresponding material and method section.
Randomization	Randomization was used for all the biological experiments.
Blinding	No blinding was done as none of the experiments described in this study involve group allocation during data collection or analyses.

Reporting for specific materials, systems and methods

We require information from authors about some types of materials, experimental systems and methods used in many studies. Here, indicate whether each material, system or method listed is relevant to your study. If you are not sure if a list item applies to your research, read the appropriate section before selecting a response.

Materials & experimental systems

n/a	Involved in the study	
☐	☒	Antibodies
☒	☐	Eukaryotic cell lines
☒	☐	Palaeontology and archaeology
☒	☐	Animals and other organisms
☒	☐	Clinical data
☒	☐	Dual use research of concern
☒	☐	Plants

Methods

n/a	Involved in the study	
☒	☐	ChIP-seq
☒	☐	Flow cytometry
☒	☐	MRI-based neuroimaging

Antibodies

Antibodies used

For western blot analyses, The primary anti-FLAG antibody (F9291, Sigma, USA) and secondary anti-Mouse antibody (DY60203, DEEYEE, China) were used to detect the expression of RNA5P-3×FLAG fusion proteins. Detection with a primary anti-Tub2 β-tubulin antibody (HA720035, HUABIO, China) and secondary anti-Rabbit antibody (DY60202, DEEYEE, China) were used as the loading control. A dilution of 1:1,000 is used for primary antibodies and 1:10,000 or secondary antibodies.

Validation

All antibodies utilized in this study are commercially sourced and have undergone validation. These antibodies have been also validated for their efficacy in detecting proteins associated with Fusarium graminearum in prior investigations (references: https://doi.org/10.1371/journal.pgen.1009185; https://doi.org/10.1016/j.fgb.2019.103251).

Plants

Seed stocks

N/A

Novel plant genotypes

N/A

Authentication

N/A
